# High Glucose Accelerates Tumor Progression by Regulating MEDAG-Mediated Autophagy Levels in Breast Cancer

**DOI:** 10.7150/ijbs.70002

**Published:** 2022-07-04

**Authors:** Chenyuan Li, Si Sun, Yi Tu, Hanpu Zhang, Feng Yao, Shichong Liao, Shengrong Sun, Zhiyu Li, Zhong Wang

**Affiliations:** 1 Department of Breast and Thyroid Surgery, Renmin Hospital of Wuhan University, Wuhan, Hubei, P. R. China.; 2 Department of Clinical Laboratory, Renmin Hospital of Wuhan University, Wuhan, Hubei, P. R. China.

**Keywords:** breast cancer, diabetes, MEDAG, EMT, AMPK signaling

## Abstract

Recent studies have shown that diabetes is a major risk factor for breast cancer (BC), but the mechanism is incompletely understood. Mesenteric estrogen-dependent adipogenesis (MEDAG) plays a significant role in both glucose uptake and BC development. However, the relationship between MEDAG and BC under high glucose (HG) conditions remains unclear. In our study, MEDAG expression was higher in BC tissue from diabetic patients than in BC tissue from nondiabetic patients. HG promoted BC progression *in vitro* and *in vivo* by upregulating MEDAG expression. Furthermore, MEDAG deficiency increased the autophagosome number and autophagic flux. Moreover, inhibition of autophagy partially reversed MEDAG knockdown (MEDAG^KD^)-induced suppression of tumorigenic biological behaviors and epithelial-mesenchymal transition (EMT) progression. Finally, MEDAG significantly suppressed AMPK phosphorylation. Additionally, the AMPK inhibitor Compound C markedly reduced autophagosome accumulation and antitumor effects in MEDAG^KD^ cells. Treatment with the AMPK activator AICAR exhibited similar effects in MEDAG-overexpressing (MEDAG^OE^) cells. In conclusion, the MEDAG-AMPK-autophagy axis is vital to BC progression in diabetic patients. Our findings provide a novel treatment target for BC in patients with diabetes.

## Introduction

Breast cancer (BC) is the most prevalent neoplasm among women and is the leading cause of cancer death in females 1, 2. The worldwide incidence and mortality of BC in females have almost doubled in the past 30 years 2. The latest study revealed that 18% of patients with BC have previously been diagnosed with diabetes 3. Diabetes is linked to highly aggressive BCs. Specifically, the risk of death from BC is increased by 24-44% in diabetic women 3. However, the underlying mechanism is unclear.

Mesenteric estrogen-dependent adipogenesis (MEDAG) participates in processes that promote adipocyte differentiation, lipid accumulation, and glucose uptake in mature adipocytes 4, 5. A recent study demonstrated that the MEDAG expression level was positively correlated with the hemoglobin A1c level and that MEDAG was overexpressed in the pancreatic islet tissue of type 2 diabetes mellitus (T2DM) patients. They concluded that MEDAG was a novel hub candidate for T2DM treatment 6. On the other hand, MEDAG was reported to be overexpressed in meningioma 7 and papillary thyroid microcarcinoma, and its overexpression was related to lymphatic metastasis and poor prognosis 8. Our previous study showed that MEDAG promoted epithelial-mesenchymal transition (EMT) in BC via the AKT/AMPK/mTOR pathway and reduced chemosensitivity in BC cells 9. Given the critical role of MEDAG in both diabetes and tumor progression, MEDAG might be a promising therapeutic target for BC in diabetic patients.

Autophagy is a lysosome-mediated process by which nonessential or damaged cellular constituents are degraded. Physiological autophagy functions as an adaptive response to stress, ensuring the delivery of metabolic substrates to cells to satisfy their energy demands and support their growth and survival 10. Under stimulation with high glucose (HG), the 5' AMP-activated protein kinase (AMPK) pathway, which is a classical pathway for autophagy activation, is strongly suppressed 11. Furthermore, deficiency of autophagy due to diabetes is likely to aggravate BC development. Many studies have claimed that impaired autophagy promotes BC bone metastasis 12, 13. However, the involvement of autophagy in MEDAG-mediated BC progression needs further exploration.

EMT is considered the major factor in cancer metastasis and progression. During EMT, adhesion junctions and cell polarity are impaired, and tumor cells acquire mesenchymal characteristics and enhanced motility 14. Accumulating studies have revealed that there is a complex relationship between autophagy and EMT. Autophagy can support the viability of potential metastatic tumor cells but can also prevent EMT and inhibit the EMT phenotype 15, 16. Moreover, autophagy and EMT share numerous signaling pathways 14. For instance, in response to changes in intracellular energy, AMPK induces the classical autophagic process 17. In addition, AMPK signaling suppresses EMT by inhibiting Smad2/3 and TGF-β activity 18.

In this context, we revealed the possible molecular mechanism underlying the relationship between diabetes and BC. We hypothesized that MEDAG mediates EMT via AMPK-regulated autophagy under HG conditions. This research might be useful in strategies for managing BC, and MEDAG could be a potential therapeutic target for BC in patients with diabetes.

## Materials and Methods

### Cell culture and reagents

Human BC cell lines (MCF7 and MDA-MB-231) were cultured in low-glucose (5.5 mM) Dulbecco's modified Eagle's medium (DMEM; Gibco) containing 10% fetal bovine serum (FBS) (ScienCell Research Laboratories, Inc., Carlsbad, CA, USA) and 1% penicillin/streptomycin (Invitrogen; Thermo Fisher Scientific, Inc., Waltham, MA, USA) at 37 °C in a humidified atmosphere containing 5% CO_2_. Compound C (CC) was purchased from MedChemExpress, and chloroquine (CQ) was purchased from Sigma-Aldrich Corporation; both were prepared according to the corresponding instructions.

### siRNA transfection and viral infection

To inhibit the expression of MEDAG, we designed a MEDAG siRNA (5'-GCAGUUUCUCUGACCGAAATT-3') and a scrambled siRNA (5'-UUCUCCGAACGUGUCACGUTT-3') 9. These constructs were purchased from GenePharma Co. (Shanghai, China). The Flag-MEDAG and Flag-NC overexpression plasmids were obtained from GeneChem Co. (Shanghai, China). RNAiMAX and Lipofectamine 3000 (Invitrogen, USA) were used, and transfection was conducted according to the instructions.

The human MEDAG-targeting shRNA and the corresponding control shRNA lentiviruses (LV-shMEDAG and LV-shCtrl, respectively) and the GFP-mCherry-LC3B and GFP-LC3B lentiviruses (LV-mCherry-LC3B and LV-GFP-LC3B, respectively) were purchased from GeneChem Co. (Shanghai, China).

### Cell growth, migration and invasion assays

Cell growth was evaluated by a CCK-8 assay (Biosharp, BS350B). Cells were seeded in 96-well plates at a density of 2000 cells/well. After appropriate treatments, 10 μL of CCK-8 reagent was added to each well and incubated at 37 °C for 2 h. The absorbance was measured at 450 nm in a microplate reader.

Transwell assays with or without Matrigel were performed to measure cell invasion and migration, respectively. A total of 4×10^4^ cells in serum-free medium were seeded in the upper compartments of Transwell chambers, and DMEM supplemented with 10% FBS was added to the lower compartments. After 24 h, cells were fixed and stained with 0.1% crystal violet.

### Western blot analysis and antibodies

Total protein lysates were prepared and Western blot analysis was performed as previously described 19. Proteins were extracted in NP40 lysis buffer on ice. After separation by SDS-PAGE, proteins were transferred onto nitrocellulose (NC) membranes, which were blocked with 5% nonfat milk. The membranes were incubated first with primary antibodies at 4 °C overnight and then with secondary antibodies for 1 h at room temperature. Immunoreactivity was imaged using an Odyssey Infrared Imager (Li-COR Biosciences) and quantified using ImageJ software. The relevant antibodies are listed in Table S1.

### Immunohistochemistry

Immunohistochemical (IHC) staining and evaluation were performed as previously described 9. Antibodies against MEDAG (Biorbyt, orb380371), E-cadherin (E-cad; Cell Signaling Technology, 14472), N-cadherin (N-cad; Cell Signaling Technology, 13116) and Ki67 (Santa Cruz, sc-23900) were used.

### Electron microscopy

Cells were seeded in 6-well plates and transfected with the different siRNAs. After exposure to HG, the cells were fixed with precooled 2.5% glutaraldehyde for 1 h. Then, the cells were quickly removed, collected, and centrifuged for 5 min at 500 rpm. Most of the supernatant was removed, and the cells were then gently dried by blowing and allowed to sediment naturally for 1 h. The supernatant was then carefully removed, and 1 mL of new precooled 2.5% glutaraldehyde was slowly added along the tube wall. The rest of the procedure was performed as described previously 20. Digital images were acquired using an electron microscope.

### Immunofluorescence (IF) staining

After treatment, cells were fixed with paraformaldehyde and permeabilized with 0.2% Triton-X 100 for 15 min at 4 °C. After blocking with 5% BSA for 1 h, the cells were incubated with the corresponding primary antibody at 4 °C overnight. Then, they were washed with 0.05% Triton-X 100 three times and incubated with an Alexa Fluor 488-conjugated antibody (Invitrogen, A11034) for 0.5 h. Finally, nuclei were stained with DAPI. Images were acquired by laser confocal microscopy (Olympus FV31S).

### Animal experiments

All animal experiments were approved by the Animal Ethics Committee of Wuhan University. MDA-MB-231 cells with stable expression of control shRNA (shCtrl) or MEDAG shRNA were used. Streptozotocin (STZ; 80 mg/kg) was injected into four-week-old BALB/c nude mice. Five days after injection, blood glucose was measured. The mice with a fasting blood glucose concentration of greater than 11.1 mmol/L were selected as the HG group (n=6). Another 6 mice not injected with STZ were selected as the control (ctrl) group. Both the ctrl and HG groups were randomly divided into two subgroups of mice, which were injected with either shCtrl cells or shMEDAG cells (2.5×10^6^ cells per mouse) in the right iliac fossa. Twenty-five days after injection, the mice were sacrificed, and the xenografts were removed for analysis.

For the metastasis assay, the experimental groups were established as described above. ShCtrl or shMEDAG cells (1×10^6^ cells per mouse) were injected into the fourth mammary fat pad of each mouse. Approximately 5 weeks after injection, the mice were sacrificed, and their lung tissues were harvested and photographed. Hematoxylin and eosin (H&E) staining was performed to identify the presence of lung metastasis.

### Statistical analysis

Data from at least three independent experiments were analyzed, and the values are presented as the means±SDs. SPSS 22.0 was used for analysis, and GraphPad Prism 8.0 was used to generate graphs. Data are expressed as the mean±SD of three independent experiments each performed in triplicate. P<0.05 was considered statistically significant. P values<0.0001 are indicated with four asterisks (****).

## Results

### HG induces MEDAG expression and promotes the proliferation, migration and invasion of BC cells

**Figure 1:**
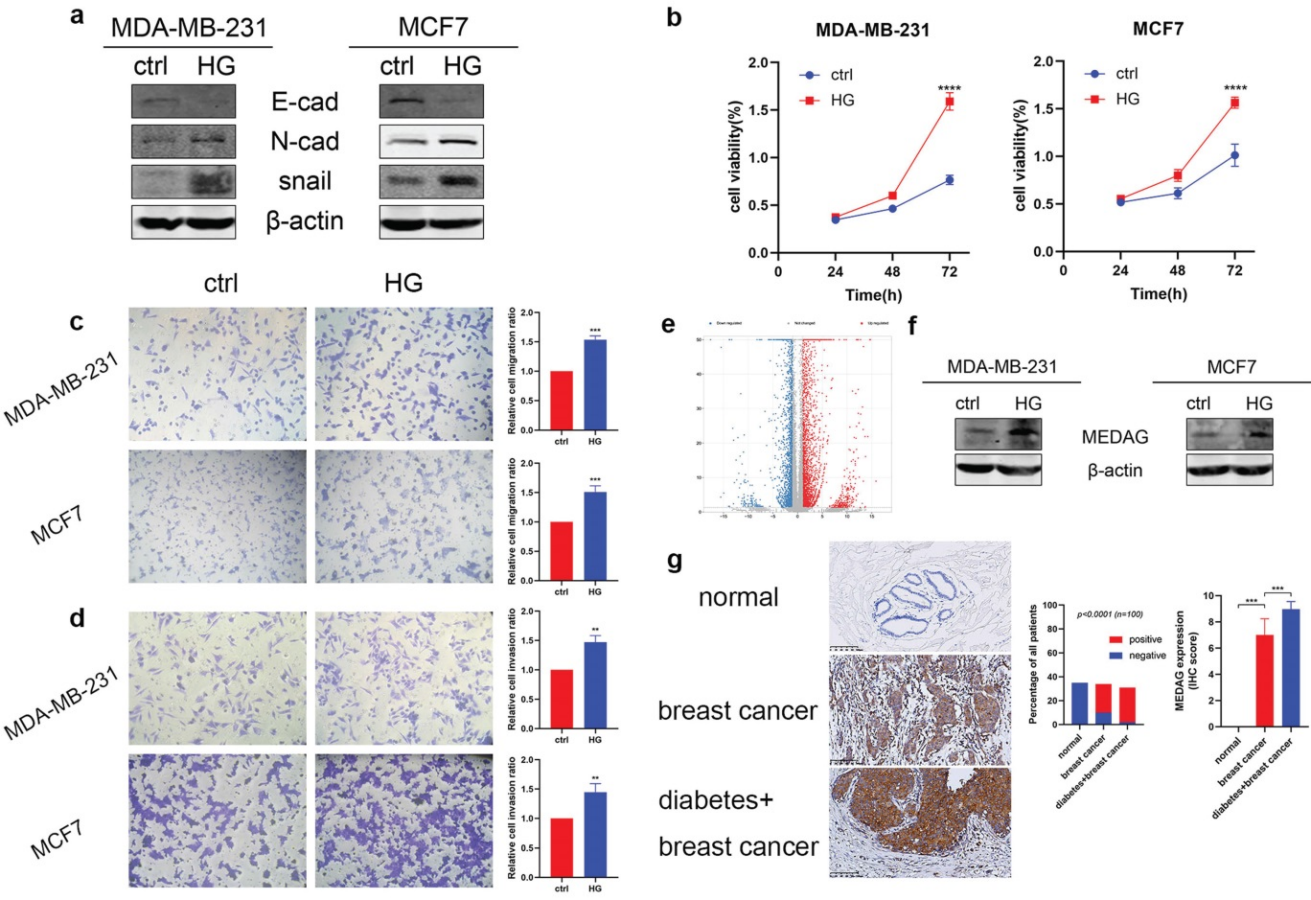
** HG promotes tumor progression and MEDAG expression in MDA-MB-231 and MCF7 cells. (a)** Cells were treated with 25 mM glucose (HG) for 24 h, and mannitol was used for comparison. The protein levels of E-cad, N-cad, snail and β-actin were measured by Western blotting. **(b)** Cells were treated as described above, and a CCK-8 assay was used to assess cell growth. **(c)** Cell migration and **(d)** cell invasion were evaluated by Transwell assays (magnification, 200×). **(e)** The volcano plot indicates differences in the upregulated and downregulated gene expression profiles between BC patients with or without diabetes. **(f)** HG-induced MEDAG expression was analyzed by Western blotting. **(g)** IHC staining of MEDAG in normal breast tissue, BC tissue from nondiabetic patients and BC tissue from diabetic patients (magnification, 200×). The proportion of each group (middle panel) and the quantitative analysis of IHC images (right panel) are shown. The values are presented as the mean ± SD of three independent experiments. **P < 0.01, ***P < 0.001, ****P < 0.0001 vs. the control group.

To verify the tumor-promoting effect of HG *in vitro*, we treated MDA-MB-231 and MCF7 cells with 25 mM glucose or with mannitol for comparison. The protein expression level of E-cad decreased, and the expression levels of N-cad and snail increased (Fig. 1a). The results of the CCK-8 assay suggested that HG promoted cell growth (Fig. 1b). Similarly, cell migration (Fig. 1c) and cell invasion (Fig. 1d) were increased in the HG environment. Furthermore, RNA-sequencing analysis between a diabetic and a non-diabetic BC patient indicated that the expression levels of MEDAG, snail, N-cad, vimentin, slug and ZEB1 tended to increase but that of AMPK tended to decrease in diabetic BC patients compared with nondiabetic BC patients (Fig. 1e). Detailed information on these genes is listed in Table S2. Western blot analysis demonstrated that HG could stimulate the expression of MEDAG (Fig. 1f). The same result was observed in tumor tissues (Fig. 1g). These results clarified that HG plays a crucial role in BC progression and regulates MEDAG expression.

### MEDAG mediates tumor progression under HG conditions

**Figure 2:**
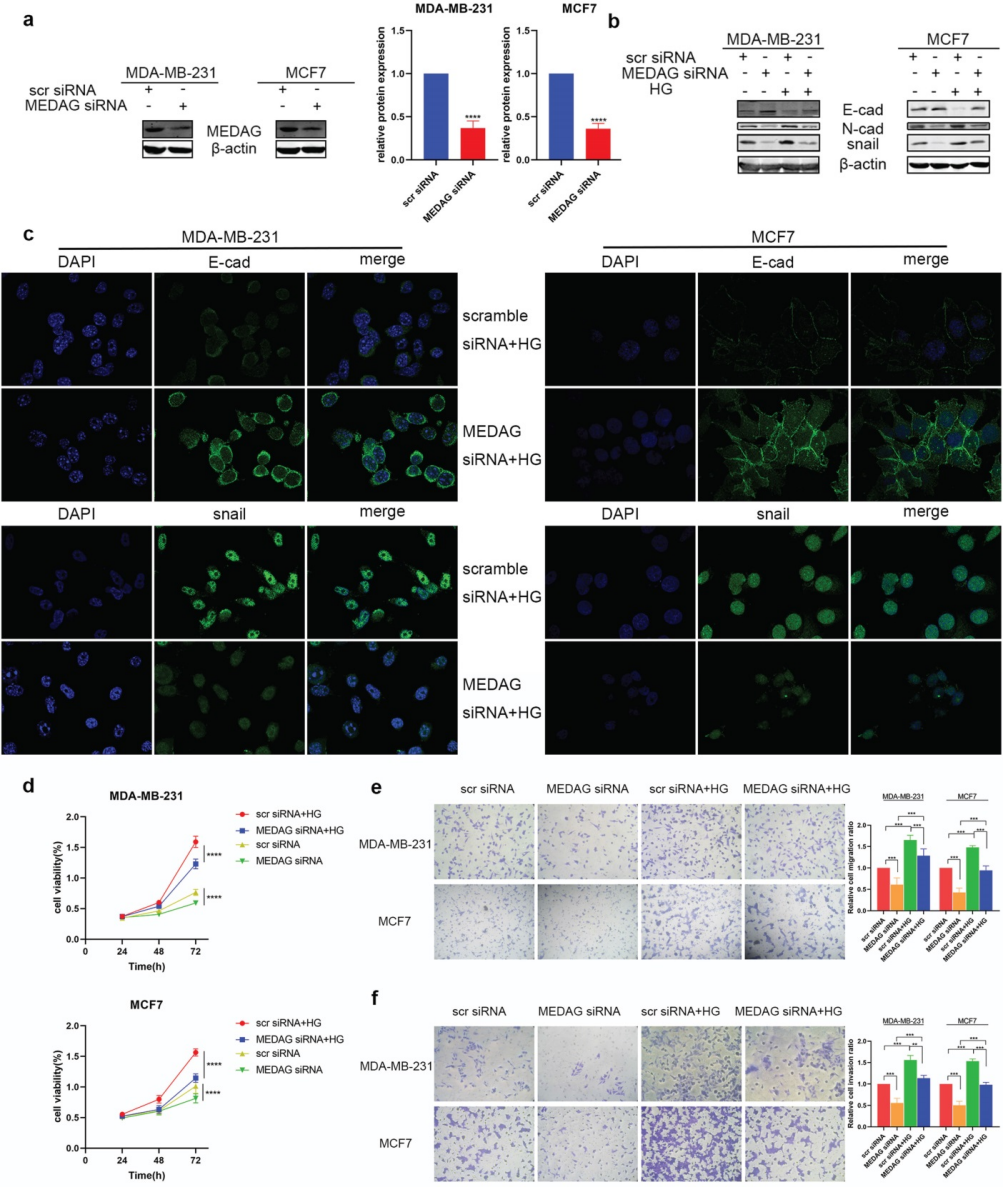
** Downregulation of MEDAG attenuates HG-induced tumor cell growth, migration and invasion. (a)** Knockdown efficiency of MEDAG in MDA-MB-231 and MCF7 cells. Cells were transiently transfected with scrambled siRNA or MEDAG siRNA for 36 h. The expression level of MEDAG was measured by Western blotting. Quantitative analysis of the ratio of the MEDAG band density compared to the β-actin band density is shown. **(b)** Expression of EMT-related proteins under normal glucose conditions and HG conditions. Cells were transiently transfected with scrambled siRNA or MEDAG siRNA for 36 h and were then treated with 25 mM glucose or with mannitol as the corresponding control for 24 h. EMT-related proteins were analyzed by Western blotting. **(c)** The expression of E-cad and snail was evaluated by IF staining. E-cad and snail are indicated by green staining, and nuclei are indicated by blue staining. **(d)** The effect of HG on cell viability after MEDAG^KD^ was evaluated. Cells were treated as described above, and cell growth was assessed by a CCK-8 assay. **(e-f)** Transwell assays of cell migration and cell invasion (magnification, 200×). The values are presented as the mean ± SD of three independent experiments. **P < 0.01, ***P < 0.001, ****P < 0.0001 vs. the control group.

MEDAG siRNA was used to downregulate the expression of MEDAG (Fig. 2a). MEDAG knockdown (MEDAG^KD^) resulted in increased E-cad expression and decreased N-cad and snail expression (Fig. 2b). In addition, the IF staining results demonstrated that under HG conditions, the expression of E-cad increased but the expression of snail decreased in MEDAG^KD^ cells (Fig. 2c). Measurement of cell growth by a CCK-8 assay showed that the viability of MEDAG^KD^ cells was decreased compared with that of cells transfected with the scrambled siRNA (Fig. 2d). In addition, the Transwell assays showed that both cell migration and invasion were decreased with MEDAG^KD^ (Fig. 2e-f).

**Figure 3:**
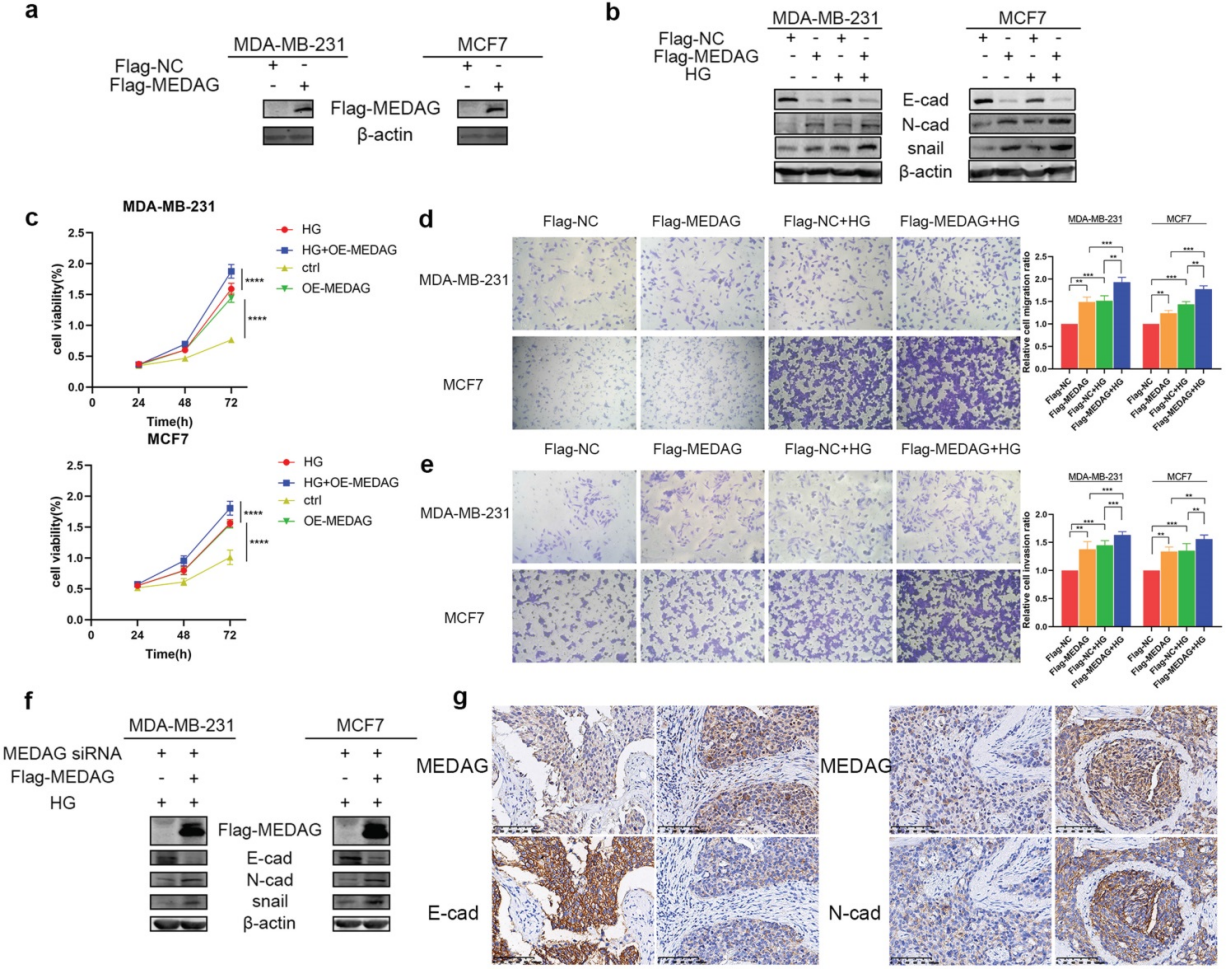
** Upregulation of MEDAG enhances HG-induced tumor cell growth, migration and invasion. (a)** MEDAG overexpression efficiency in MDA-MB-231 and MCF7 cells. Cells were transiently transfected with FLAG-NC or FLAG-MEDAG for 36 h. The expression level of Flag-MEDAG was measured by Western blotting. **(b)** Expression of EMT-related proteins in MEDAG^OE^ cells and their counterparts under normal glucose conditions and HG conditions. **(c)** Cell growth after MEDAG overexpression was assessed by a CCK-8 assay. **(d-e)** Transwell assays of cell migration and cell invasion (magnification, 200×). **(f)** MEDAG^KD^ cells were cultured in HG medium and transfected with Flag-MEDAG for 36 h. EMT-related proteins were analyzed by Western blotting. **(g)** IHC staining of MEDAG, E-cad and N-cad in tumor tissues (magnification, 200×). The values are presented as the mean ± SD of three independent experiments. **P < 0.01, ***P < 0.001, ****P < 0.0001 vs. the control group.

To further verify our hypothesis, we overexpressed MEDAG (Fig. 3a) and performed that same experiments outlined above. The expression trends of EMT-related proteins were opposite those observed in MEDAG^KD^ cells (Fig. 3b). Cell viability was increased by overexpression of MEDAG compared with that of the corresponding negative control cells (Fig. 3c). In addition, cell migration and invasion were increased (Fig. 3d-e). Furthermore, the rescue experiment showed that MEDAG overexpression in MEDAG^KD^ cells reversed inhibition of EMT induced by MEDAG^KD^ (Fig. 3f). Consistent with this finding, IHC staining of BC samples indicated that the expression of MEDAG was negatively correlated with that of E-cad and positively correlated with that of N-cad (Fig. 3g). Taken together, these results demonstrated that MEDAG is involved in the regulation of BC progression by HG.

### Inhibition of MEDAG suppresses tumor growth and lung metastasis *in vivo*

**Figure 4:**
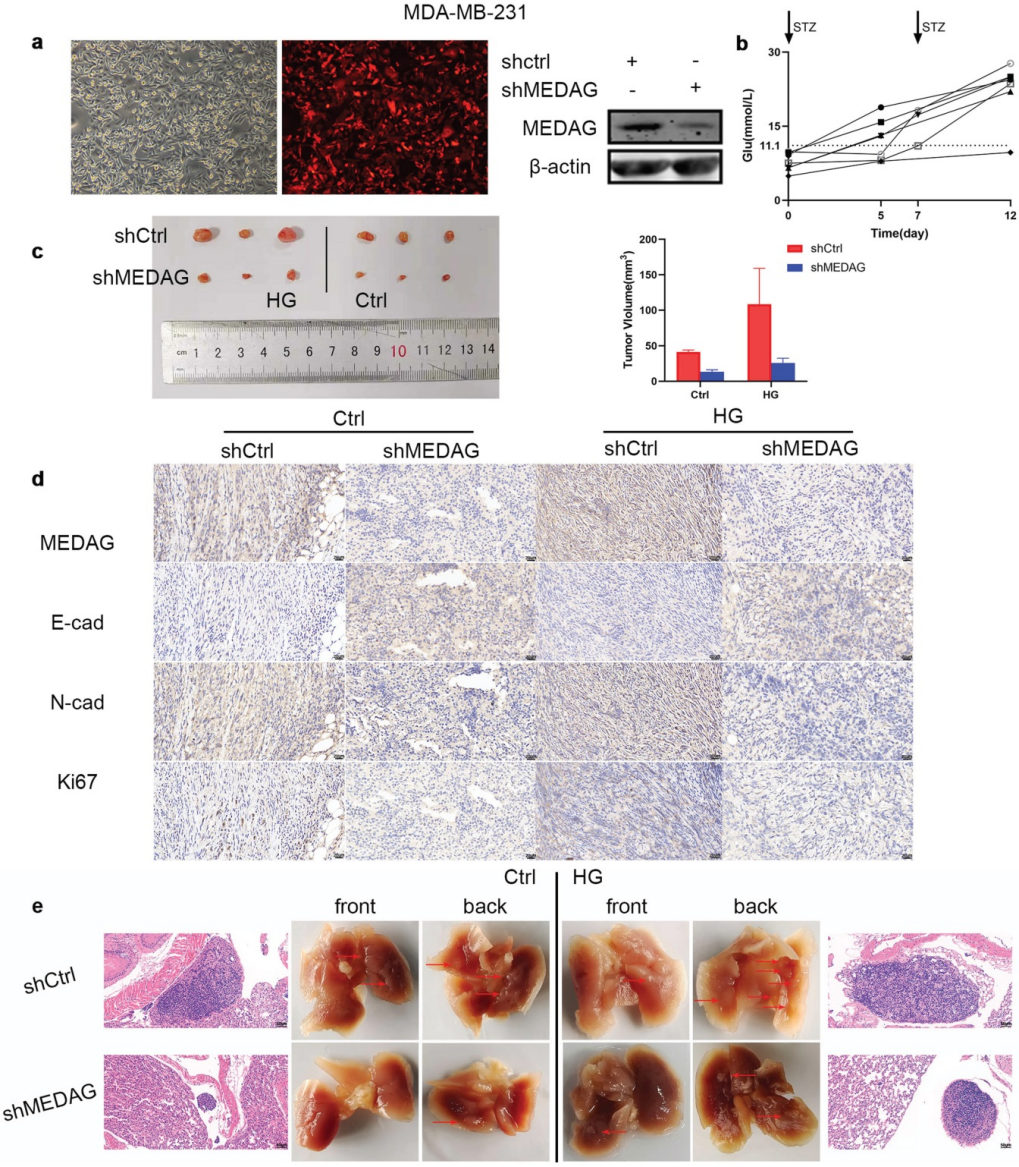
** Knockdown of MEDAG impairs tumor growth *in vivo*. (a)** MEDAG^KD^ efficiency in MDA-MB-231 cells (magnification, 100×). **(b)** Fasting blood glucose concentration after STZ injection. **(c)** Macroscopic images of tumors from the normal glucose+shCtrl, normal glucose+shMEDAG, HG+shCtrl and HG+shMEDAG groups (n=3). **(d)** IHC staining of EMT-related markers and Ki67 in xenograft tumors (magnification, 400×). **(e)** Brightfield and H&E images of lung metastases (magnification, 200×).

Based on the *in vitro* results, we established xenograft models *in vivo*. MDA-MB-231 cells were infected with LV-shCtrl and LV-shMEDAG (Fig. 4a). Mice were injected with STZ (80 mg/kg) to establish the diabetes model. Fasting blood glucose was measured before and 5 days after the injection (Fig. 4b). Mice with a blood glucose concentration of greater than 11.1 mmol/L (n=6) were selected as the HG group and randomly divided into two subgroups, and the mice in these subgroups were injected with either shCtrl (n=3) or shMEDAG (n=3) in the right iliac fossa. Mice in the ctrl group (n=6), whose blood glucose concentrations were normal, were treated as described above. Twenty-five days after the injection, we excised and photographed the transplanted tumors. The volume of tumors formed from shMEDAG cells was significantly decreased compared with that of tumors formed from shCtrl cells (Fig. 4c). IHC staining was performed, and the results were consistent with the *in vitro* results (Fig. 4d). In addition, the reduction in the metastatic ability induced by MEDAG interference was verified* in vivo*. Brightfield imaging showed increased lung metastasis in diabetic mice compared with nondiabetic mice, while MEDAG knockdown decreased the number of metastatic nodules in both diabetic and nondiabetic mice. The results of H&E staining further supported this finding (Fig. 4e). Collectively, these results indicated that downregulation of MEDAG attenuated tumor growth and lung metastasis *in vivo*.

### Autophagy mediates the modulation of tumor progression by MEDAG in a HG environment

**Figure 5:**
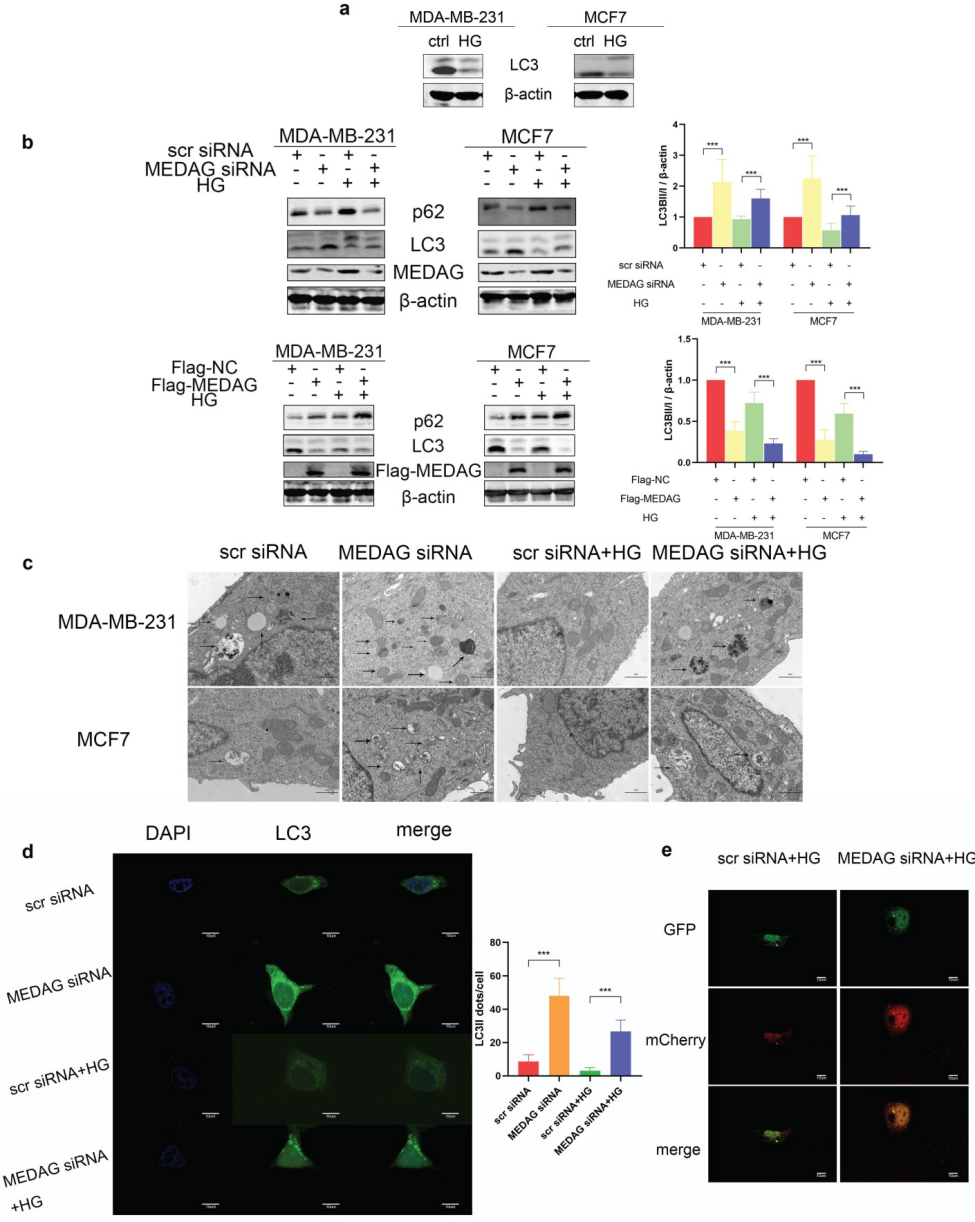
** Autophagy is activated by MEDAG^KD^. (a)** Protein level of LC3 in MDA-MB-231 and MCF7 cells under HG conditions. **(b)** Expression of autophagy-related proteins after MEDAG knockdown and overexpression under normal glucose conditions and HG conditions. **(c)** Cells were treated with scrambled siRNA or MEDAG siRNA. Autophagosomes were examined by electron microscopy at 80,000× magnification in cells with or without HG treatment. The arrows indicate autophagosomes. **(d)** LC3 puncta were detected by confocal microscopy. Green fluorescence indicates LC3, and blue fluorescence indicates nuclei. **(e)** Under HG conditions, MEDAG^KD^ cells were infected with LV-GFPLC3B and LV-mCherry-LC3B. Autophagic flux can be observed. The red dots indicate autolysosomes, and the yellow dots indicate autophagosomes. The values are presented as the mean ± SD of three independent experiments. **P < 0.01, ***P < 0.001, ****P < 0.0001 vs. the control group.

Autophagy has been reported to be involved in HG-regulated BC progression 21. Therefore, we determined the autophagy level in HG-treated BC cell lines and found that the levels of both LC3II and LC3I were significantly decreased after HG treatment (Fig. 5a). To evaluate whether MEDAG is associated with autophagy, MEDAG siRNA and Flag-MEDAG were transfected into BC cell lines. MEDAG^KD^ decreased the expression of p62 and increased the levels of both LC3-Ⅱ and LC3-I, while MEDAG overexpression resulted in the opposite changes (Fig. 5b). Similar effects were observed by electron microscopy, which showed more autophagosome structures in MEDAG^KD^ cells than in scrambled siRNA-transfected control cells (Fig. 5c). Furthermore, MDA-MB-231 cells were simultaneously infected with LV-GFP-LC3B and transfected with MEDAG siRNA. The number of LC3 puncta clearly increased upon MEDAG^KD^ (Fig. 5d). To further confirm that autophagic flux was promoted by MEDAG siRNA, we also infected MDA-MB-231 cells with LV-GFP-LC3B and LV-mCherry-LC3B. Under HG conditions, MEDAG^KD^ cells showed a decrease in green fluorescence intensity representing LC3B and an increase in red fluorescence intensity (Fig. 5e).

**Figure 6:**
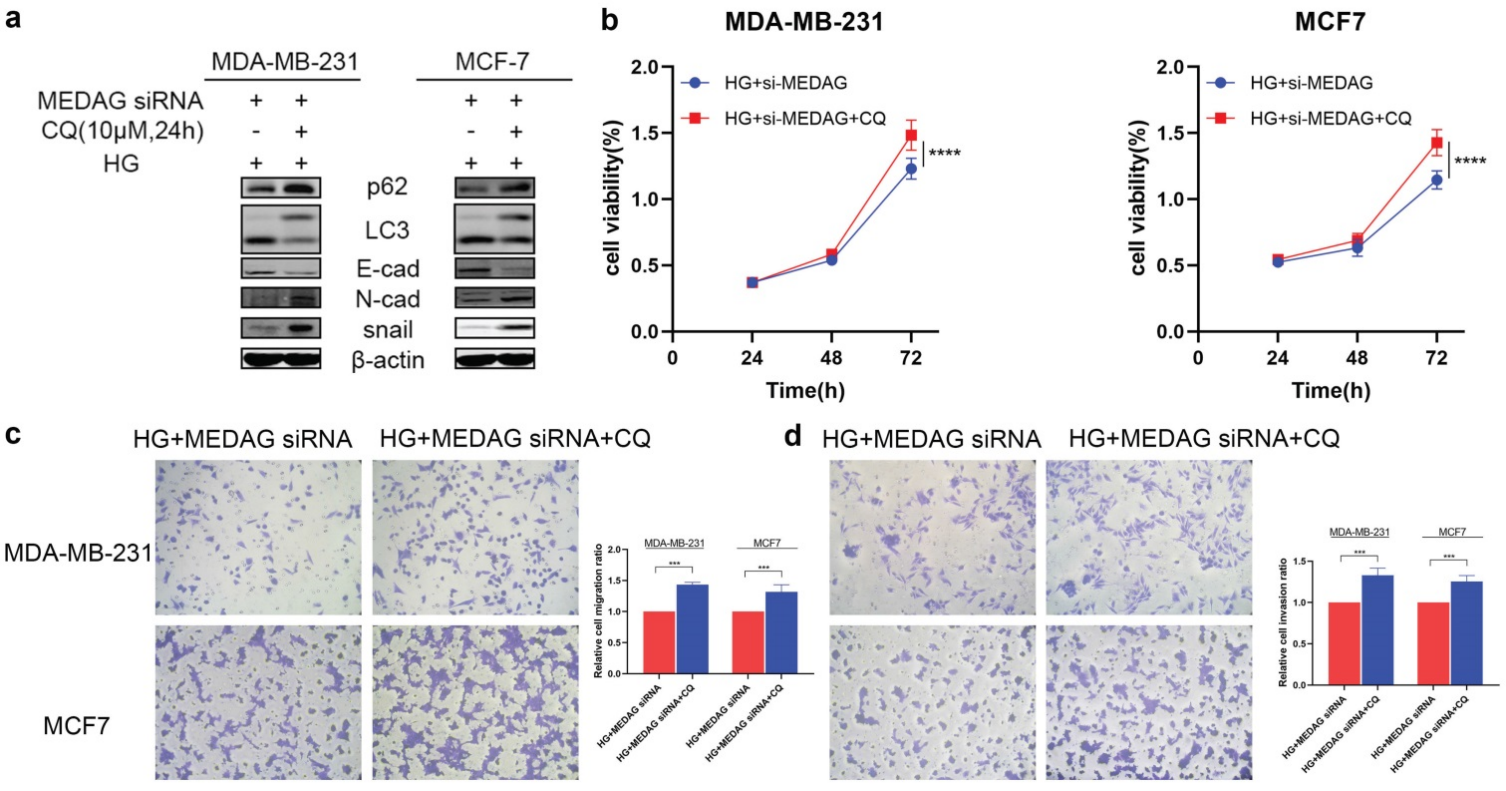
** Inhibition of autophagy promotes the growth, migration and invasion of MDA-MB-231 and MCF7 cells. (a)** MEDAG^KD^ cells were treated with the autophagy inhibitor CQ (10 µM) for 24 h under HG conditions. EMT-related proteins were analyzed by Western blotting. **(b)** Cell growth was measured by a CCK-8 assay. **(c, d)** Cells were treated as described above. Cell migration and invasion were evaluated by Transwell assays. The values are presented as the mean ± SD of three independent experiments. **P < 0.01, ***P < 0.001, ****P < 0.0001 vs. the control group.

We then inhibited autophagy with CQ and assessed tumor progression. The results of Western blot analysis, CCK-8 assays and Transwell assays demonstrated that inhibition of autophagy reversed the reduction in EMT induced by MEDAG siRNA (Fig. 6). These data strongly demonstrated that inhibition of autophagy can reduce the inhibitory effect of MEDAG^KD^ on malignant behaviors of cells induced by a HG environment.

### MEDAG regulates BC progression via the AMPK pathway under HG conditions

**Figure 7:**
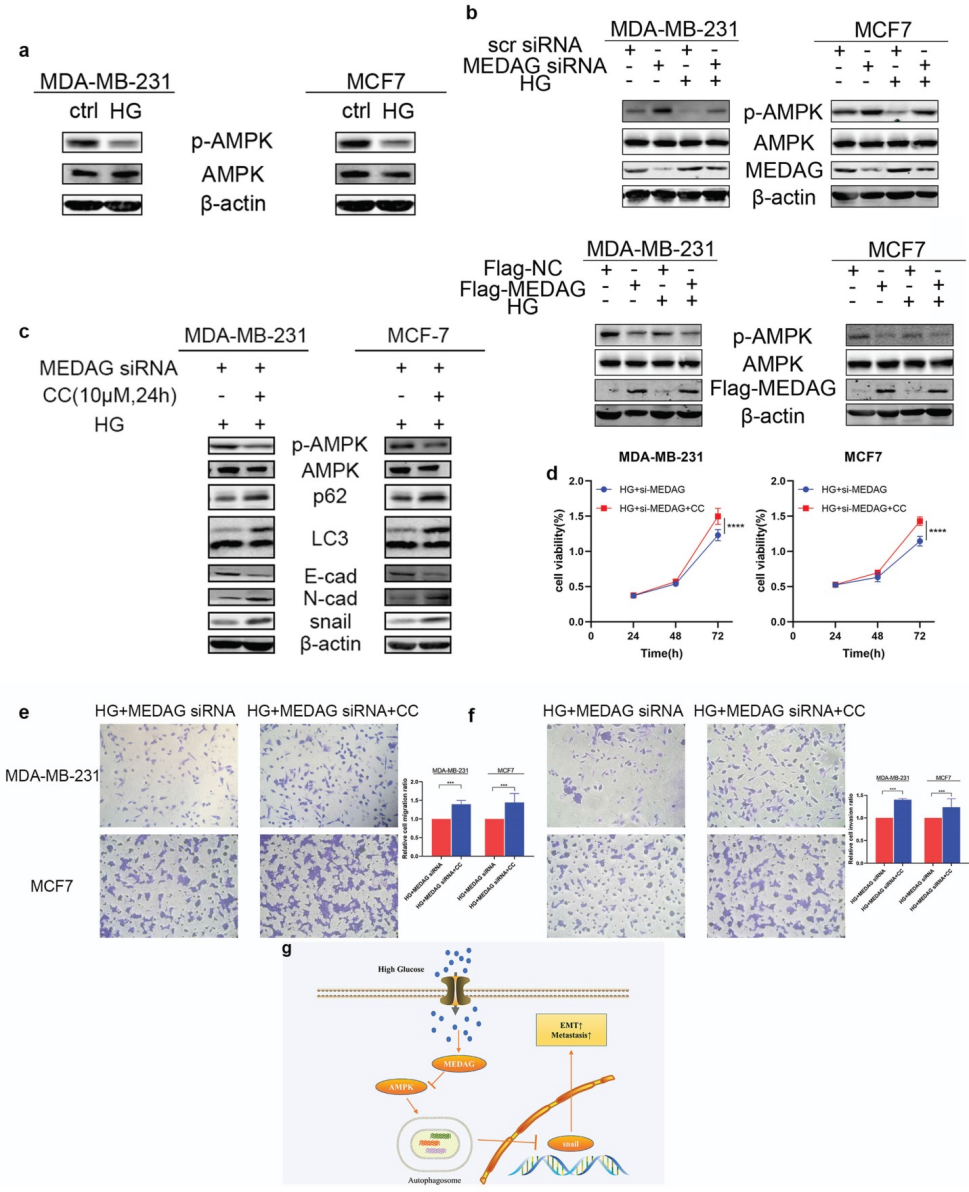
** MEDAG mediates EMT through the AMPK pathway under HG conditions. (a)** Protein levels of p-AMPK and AMPK after culture under normal glucose and HG conditions. **(b)** Protein levels of p-AMPK and AMPK in MEDAG^KD^ and MEDAG^OE^ cells in a normal glucose environment and a HG environment. **(c)** MEDAG^KD^ cells were treated with the AMPK inhibitor CC (10 µM) for 24 h under HG conditions. EMT-related proteins were analyzed by Western blotting. **(d)** A CCK-8 assay was performed to evaluate the growth of MEDAG^KD^ cells after CC treatment under HG conditions. **(e, f)** Cell migration and invasion after CC treatment in MEDAG^KD^ cells under HG conditions. **(g)** Schematic diagram showing the mechanism by which MEDAG promotes BC progression in a HG environment. The values are presented as the mean ± SD of three independent experiments. **P < 0.01, ***P < 0.001, ****P < 0.0001 vs. the control group.

Given that the AMPK pathway is the classical signaling pathway of EMT and autophagy in response to glucose concentration, we next verified the involvement of AMPK in the progression of MEDAG-mediated EMT. HG stimulation clearly reduced the levels of both p-AMPK and total AMPK (Fig. 7a). The levels of both p-AMPK and total AMPK increased after MEDAG^KD^ but decreased after MEDAG overexpression (Fig. 7b). Under HG conditions, MEDAG^KD^ cells were treated with the AMPK inhibitor CC. The expression of E-cad decreased but that of N-cad and snail increased compared with those in MEDAG^KD^ cells without CC treatment (Fig. 7c). Additionally, the CCK-8 and Transwell assays showed that AMPK inhibition reversed the decreases in cell growth, migration and invasion (Fig. 7d-f). What's more, AMPK activator AICAR exhibited similar effects in MEDAG-overexpressing (MEDAG^OE^) cells (Fig. S1). Taken together, these results suggested that the AMPK pathway is involved in MEDAG-mediated BC progression. Therefore, we concluded that MEDAG upregulation by HG inhibits the AMPK-autophagy axis, thereby promoting EMT progression and favoring tumorigenic biological behaviors (Fig. 7g).

## Discussion

Here, we provided evidence supporting the role of MEDAG in regulating BC progression in diabetic patients. We found that MEDAG was highly expressed in BC patients with diabetes compared with nondiabetic BC patients. *In vitro*, HG stimulated the expression of MEDAG, thereby promoting BC development. The *in vivo* experiments also indicated that MEDAG deficiency suppressed tumor growth and metastasis in both diabetic and nondiabetic mice. Further exploration showed that HG-induced MEDAG promoted EMT progression by inhibiting the AMPK-autophagy axis. Therefore, MEDAG is a potential therapeutic target for BC in patients with diabetes.

Recent studies have provided evidence that HG induces aggressiveness in cancers and is useful for predicting cancer progression 22. HG conditions have been reported to induce cancer cell proliferation, angiogenesis and chemoresistance in BC, pancreatic cancer, gastrointestinal cancers and genitourinary tract cancers 23-26. Additionally, a correlation between HG conditions and MEDAG expression has been reported. The authors claimed that MEDAG is expressed in pancreatic islets and is essential for T2DM 6. Furthermore, based on our previous study, which showed that MEDAG promotes EMT and reduces epirubicin sensitivity through the Akt/AMPK/mTOR axis in BC 9, we hypothesized that MEDAG is involved in HG-induced BC progression. Therefore, we evaluated MEDAG expression in HG-treated BC cell lines and observed that MEDAG was upregulated by HG treatment. Additionally, under HG conditions, MEDAG interference inhibited EMT progression and tumorigenic biological behaviors, but MEDAG overexpression promoted them. Therefore, our results showed the underlying mechanism by which HG promotes tumor progression via upregulation of MEDAG.

Reduced levels of autophagy after HG treatment have been observed in pancreatic cancer and BC 23, 24. Autophagy has also been shown to be induced by glucose starvation in glioblastoma cells 27. These results are consistent with those of our study; specifically, we found that the autophagy level was reduced by HG treatment. However, the role of autophagy in the HG environment during cancer development remains unclear. Lack of glucose was found to induce autophagy in BC cells and favor cell survival compared to that of HG-treated cells 21. In contrast, autophagy inhibition reversed HG-induced EMT in lung cancer cells 28. In our study, autophagy inhibition after HG treatment contributed to tumor growth, migration and invasion. These differences in the effects of autophagy in a HG environment may be due to cellular diversity. Furthermore, our study explored the mechanism by which HG conditions decrease the level of autophagy. MEDAG deficiency in HG-treated BC cells significantly increased the number of autophagosomes and promoted autophagic flux. More interestingly, treatment with an autophagy inhibitor partially reversed MEDAG^KD^-mediated inhibition of tumor progression under HG conditions. Thus, MEDAG-mediated autophagy is involved in HG-induced BC progression.

It is reported that the AMPK pathway is downregulated in response to HG environments 29. Moreover, the AMPK pathway is a classical autophagy-inducing signaling pathway. AMPK can promote autophagy by directly activating Ulk1 through phosphorylation at Ser317 and Ser777 29. In a HG environment, high mTOR activity prevents Ulk1 activation by disrupting the interaction between Ulk1 and AMPK 29. In addition, our previous study showed that MEDAG mediates autophagy via the AMPK pathway 9. Therefore, we further evaluated AMPK expression in MEDAG^KD^ and MEDAG-overexpressing (MEDAG^OE^) cells under HG conditions. Our study demonstrated that MEDAG participates in HG-mediated suppression of the AMPK pathway. Moreover, AMPK inhibition favored the development of BC from HG-treated MEDAG^KD^ cells.

In conclusion, our findings suggest that HG-stimulated MEDAG inhibits the AMPK-autophagy axis, thus promoting EMT progression and BC development. Therefore, MEDAG is a new potential therapeutic target for BC, especially in BC patients with diabetes.

However, more work is needed to further investigate the mechanisms by which HG induces MEDAG expression. Whether this effect is mediated by transcriptional regulation or involves other molecules is unknown. Furthermore, mass spectrometry is likely to be helpful for improving our understanding of how MEDAG increases the level of phosphorylated AMPK.

## Supplementary Material

Supplementary figure and tables.

## Abbreviations

BC: breast cancer
MEDAG: mesenteric estrogen-dependent adipogenesis
HG: high glucose
EMT: epithelial-mesenchymal transition
T2DM: type 2 diabetes mellitus
PTMC: papillary thyroid microcarcinoma
AMPK: 5' AMP-activated protein kinase
