# Supplementary material for: High Glucose Accelerates Tumor Progression by Regulating MEDAG-Mediated Autophagy Levels in Breast Cancer
Source: Int J Biol Sci. 2022 Jul 4;18(11):4289–300. doi: 10.7150/ijbs.70002 (PMC9295059; doi:10.7150/ijbs.70002)
Supplement: Supplementary file 1 — Supplementary figure and tables. [file ijbsv18p4289s1.pdf]

Table S1. Primary antibodies and secondary antibodies for Western blotting

Antibody	Concentration	Article number	Company
MEDAG	1:500	orb326838	Biorbyt
E-cadherin	1:200	sc-7870	Santa Cruz
N-cadherin	1：1000	13116	Cell Signaling Technology
Snail	1：1000	3879	Cell Signaling Technology
p-AMPK	1：1000	2535	Cell Signaling Technology
AMPK	1：1000	5831	Cell Signaling Technology
P62	1：2000	P0067	Sigma Aldrich
LC3	1：5000	M186-3	Medical & Biological Laboratories
FLAG	1：5000	F1804	Sigma
β-Actin	1：10000	A5441	Sigma
Anti-rabbit IgG (H+L)	1：10000	5151	Cell Signaling Technology
Anti-mouse IgG (H+L)	1：10000	5257	Cell Signaling Technology

Table S2. The detailed information of some gene expression

Gene ID	Symbol	Log2 ration (with/without)	Up or Down	P-value
84935	MEDAG	2.31410859	Up	1.14E-17
6615	Snail	0.195071815	Up	0.415388
6591	Slug	2.180054687	Up	4.47E-35
7431	vimentin	1.40356788	Up	0
6935	ZEB1	0.944735981	Up	4.93E-11
64403	N-cadherin	0.147990031	Up	0.586994
5562	AMPK	-0.116661534	Down	0.23801

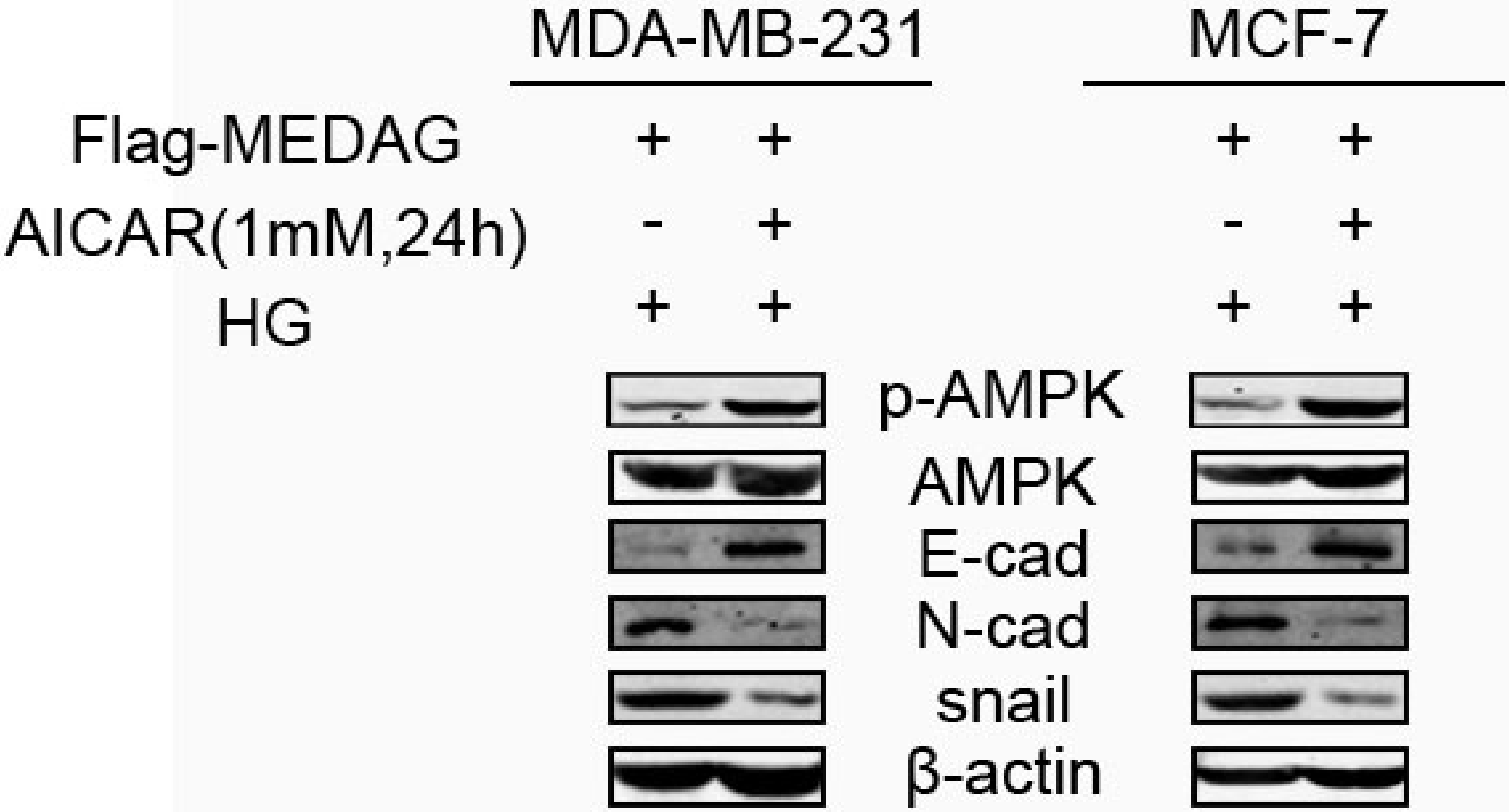
MDA-MB-231
MCF-7
Flag-MEDAG
AICAR(1mM,24h)
HG
+ +
- +
+ +
+ +
- +
+ +
p-AMPK
AMPK
E-cad
N-cad
snail
β-actin
